# Anti-triple-negative breast cancer metastasis efficacy and molecular mechanism of the STING agonist for innate immune pathway

**DOI:** 10.1080/07853890.2023.2210845

**Published:** 2023-05-10

**Authors:** Xing Lu, Xiang Wang, Hao Cheng, Xiaoqing Wang, Chang Liu, Xiangshi Tan

**Affiliations:** Department of Chemistry & Institutes of Biomedical Sciences, Fudan University, Shanghai, China

**Keywords:** Metalloenzyme cGAS, cGAMP, STING pathway, metastatic breast cancer, EMT, PI3K/AKT pathways, tumour icroenvironment

## Abstract

**Background:**

With high recurrence and metastatic rates, triple-negative breast cancer (TNBC) has few therapy choices. The innate immune stimulator of interferon genes protein (STING) pathway has emerged as a critical foundation for improving anticancer immunotherapy. Although 2’,3’-cGAMP has been shown to have therapeutic potential as a STING agonist in subcutaneous solid tumour treatments in mice, the effect of cGAMP in metastatic malignancies has received less attention.

**Methods:**

Bioluminescence imaging technology was applied to monitor TNBC tumour cell metastasis in living mice. Serum biochemical test and blood routine examination of mice were used to demonstrate cGAMP administration had no toxicity. The activation of DCs and CD8+ T cells was demonstrated by flow cytometry. The pharmacological mechanism of cGAMP for suppressing breast tumour metastasis was also explored.

**Results:**

cGAMP treatment substantially suppressed tumour development and metastasis without adverse effects. cGAMP activated the cGAS-STING-IRF3 pathway, which modified the tumour immune milieu to reverse the Epithelial-Mesenchymal Transition (EMT) and PI3K/AKT pathways and prevent tumour metastasis. It was postulated and proven that cGAMP had a pharmacological mechanism for reducing breast tumour metastasis.

**Conclusion:**

The findings suggest that cGAMP could be useful in the immunotherapy of immune-insensitive metastatic breast cancer.

## 1. Introduction

Cancers have a high mortality and morbidity rate. Breast cancer (BC) is the most common malignant cancer in females worldwide, and metastasis is the leading cause of death [1]. Patients are classified into ER/PR+, HER2+, or triple-negative subtypes based on the expression of the oestrogen receptor (ER), human epidermal growth factor receptor 2 (HER2) and progesterone receptor (PR) [2]. Among them, 20% of BC cases are triple-negative breast cancers (TNBC) that exhibit a high rate of recurrence, poor prognosis, a strong ability to metastasize and low survival rate. Because of the negative expression of hormone receptors and HER2, TNBC is less responsive to mature endocrine therapy and targeted therapy than other types of BC. Moreover, the local recurrence rate and regional lymph node metastasis rate of TNBC patients after surgical treatment are higher than those of non-TNBC patients, and treatment options for TNBC are limited [3]. Conventionally, cisplatin and platinum-based chemotherapeutics are first-line treatment options in clinical routine [4]. However, chemotherapy has disadvantages of indiscriminate attacks on human cells, strong toxicity and noticeable side effects. With the development of studies on the biological behaviour and related signalling pathways of TNBC, immunotherapy has been a type of safe and promising therapy for cancer treatments. Monoclonal antibodies named Trastuzumab and Pertuzumab have been approved for BC treatment [5]. Checkpoint blockade is used to broaden therapeutic choice for a variety of solid tumours, such as PD-L1 or CTLA-4 blocking, however, only about one-third of patients get a strong reaction [6], advising that we urgently need to exploit alternative approaches for this disease. STING is becoming a promising therapeutic target. The cGAS-STING-IRF3 pathway has been highlighted as an important regulator of viral DNA recognition for successful host defence [7], which is issued as a powerful pathway for cancer treatments [8]. The innate immune system, which includes the stimulator of interferon genes (STING), has been identified as a key mechanism for boosting antitumour immunity *via* type I interferon (I-IFN) [9]. cGAS is a nucleic acid transferase that produces endogenous 2′,3′-cyclic guanylate-adenylate (2′,3′-cGAMP) and activates STING [10]. As an agonist of STING, cGAMP is synthesized by cGAS and binds to STING to activate the cGAS-cGAMP-STING pathway. Following that, STING recruits TBK1 to activate IRF3 inducing the proinflammatory response to enhance innate and adaptive immunity [11], which is critical in anti-microbial infection and anti-tumour activities [12]. At the same time, cGAMP induces the expression of pro-inflammatory cytokines and chemokines, leading to maturation of dendritic cells and cross-stimulation of CD8+ T cells to promote tumour killing [13]. It largely suppresses tumour cell growth while boosting tumour cell apoptosis and suppressing oncogene expression and anti-tumour angiogenesis. Metastasis is the process that tumour cells invade from primary site to lymphatic vessels, blood vessels or other sites, which further form the same type of tumour tissues as primary tumour. Metastasis is a hallmark of malignant tumours, and patients with metastasis have a low cure rate and poor prognosis. Previous research found that the proliferation and metastasis of BC are linked to several signalling pathways, and that specific cytokines in the tumour microenvironment (TME), such as CXCR4 and CXCL12, play an important role in BC metastasis [14]. The innate immune and adaptive immune system have complicated synergistic effects on regulating tumour metastasis. However, the mechanism by which STING agonists inhibit breast tumour metastasis by inducing an antitumour immune response is uncertain. The efficiency and pharmacological mechanism of cGAMP in reducing triple-negative BC metastasis were thoroughly studied in this work. We selected the 4T1-Luc cell line, which was cloned from 4T1 cells transfected with the firefly luciferase gene, to establish a tumour metastasis model. The antitumour efficacy of cGAMP in metastatic BC was evaluated. cGAMP treatment substantially inhibited BC pulmonary metastases, improving splenomegaly and pathological abnormalities caused by systemic infiltration of cancer cells. Based upon the immune cytokine expressions in mice serum and lung tissues, the immune cell expressions in spleens and the related protein regulations in PI3K/AKT signalling pathway and EMT signalling pathways, the pharmacological mechanism of cGAMP in suppressing BC pulmonary metastasis was investigated further.

## 2. Materials and methods

### 2.1. Materials and reagents

cGAMP was produced in our laboratory as previously described [15,16]. The 4T1-Luc cell lines were donated by Ruikang Tang’s lab at Zhejiang University. All of the mouse ELISA kits were provided by Biolegend (San Diego, CA, USA). Flow cytometry monoclonal antibodies were obtained from eBiosciences (San Diego, CA, USA) and analysed using a BD FACS Cablibur flow cytometer (BD Bioscience, San Jose, CA, USA). Immunofluorescence antibodies were given by Cell Signaling Technology (Boston, USA, USA). A fluorescence microscope was used to photograph the H&E stain (Leica, Germany). The leftover analytic grade solvents or compounds were used without being purified.

### 2.2. Mice and cells

The Institutional Animal Care and Use Committee at Fudan University (Shanghai) authorized all operations in the study in accordance with the Animal Experimentation Ethics Guidelines. The Beijing Vital River Laboratory Animal Technology Co. Ltd. provided us with 18 female seven-week-old specific pathogen-free (SPF) BALB/c mice weighing 18–22 g (Beijing, China). The mice were then kept in a specific pathogen-free (SPF) facility (temperature, 22 ± 2 °C; humidity, 50 ± 10%) with a 12/12-h light/dark cycle and free access to normal laboratory animal feed and tap water. During the investigation, no mice were sacrificed. The animals were killed *via* CO_2_ inhalation and cervical vertebra dislocation at the end of the trial. Respiratory and cardiac arrest, as well as a lack of a righting response, verified the animal’s death.

### 2.3. cGAS & cGAMP preparation

Mouse cGAS protein and cGAMP were made following [15,16]. The modified pET-28(a) vector was used to clone mouse cGAS. 5 μL plasmid was added into 100 μL E.coli BL21 (DE3) cells. After 30 min of ice bath, the cells were subjected to heat at 42 °C for 90s. After 10 min of ice bath again, the cells were smeared on LB plate containing antibiotic, then the plate was placed in a 37 °C incubator overnight. The monoclonal colonies grown overnight were selected and cultured in LB medium containing antibiotic at 37 °C at 200 rpm until OD value reached 0.6–0.8, then IPTG was added. The bacteria were collected after cooling culture for 12h. cGAS proteins were obtained after extracted and purified by Ni-NTA column. cGAMP was synthesized by cGAS enzymatic synthesis method with the existence of ATP, GTP and some metal salt in specified conditions. Lyophilized endotoxin-free cGAMP was stored at −20 °C for subsequent use.

### 2.4. Establishment of tumour models and treatments

In a humidified 37 °C incubator with 5% CO_2,_ 4T1-Luc cells transfected with luciferase were grown in RPMI-1640 media (Hyclone, USA) with 10% heat-inactivated FBS (GIBCO, USA) and 1% penicillin/streptomycin. 4T1-Luc cells were trypsinized and washed before resuspended in RPMI-1640 media without foetal bovine serum and antibiotics at 10^6^ cells/mL. 200 μL cells were injected intravenously into the tail vein of mice. The tumour-bearing mice were randomly separated into two groups one day following injection. One group served as a control, while the other received cGAMP (20 mg/kg) intraperitoneally daily for a month. Full blood was obtained at the end of the study for routine blood testing. Mouse blood samples were extracted from the ophthalmic vein after anaesthesia with a mixture of oxygen and isoflurane, and serum was recovered by centrifuging at 300 g for 30 min for the serum biochemical test.

### 2.5. In vivo bioluminescence imaging of animal organs

We created 4T1-Luc cell lines to express firefly luciferase stably, which allowed us to track and quantify cells *in vivo. In vivo* Biophotonic imaging was used to identify tumour cell spread and proliferation. Mice were weighed and intraperitoneally treated with 150 mg/kg luciferin. After 9 min of luciferin treatment, the animals were pre-anesthetized using an oxygen-isoflurane mixed gas (1%–3%). At 12 min following luciferin administration, the animals were placed into the imaging chamber for bioluminescence measurement assessment using a Xenogen IVIS Lumina XRMS Series III live animal bioluminescence imaging system (Perkin Elmer, USA). On the last day, mice were sacrificed, then their hind limbs and organs were imaged and removed within 10 min.

### 2.6. Flow cytometry analyses of immune cells in spleens

We removed spleens from mice and isolated spleen cells at the termination of study [17]. At the end of the experiment, we sacrificed the mice and isolated their spleens. We got single-cell suspensions from disrupted spleens by filtering them through strainers (70 μm). Splenocytes were rinsed, then resuspended using cold PBS (pH 7.4), before lysed with lysis buffer. For T cells detection, we stained splenic CD8^+^ T cells with CD8α and CD3 antibodies and stained CD4^+^ T cells utilizing CD4 and CD3 antibodies. Neutrophils were labelled by CD45^+^CD11b^+^Ly6G^+^. M2-type tumour-associated macrophages (M2-TAM) were labelled by CD45^+^CD11b^+^F4/80^+^CD206^+^. We labelled DCs through CD11c monoclonal antibodies, which we stained with anti-mouse CD80, CD86, MHC II and CD40.

### 2.7. Enzyme-linked immunosorbent assay (ELISA)

Mice blood was collected and centrifuged at 1000 rpm for 10 min to extract serum for the test. In presence of protease inhibitors, lung tissues from three groups of uniform weight were mechanically homogenized. Supernatant homogenates were produced after centrifugation and kept at −80 °C for further use. The concentration of cytokines in the serum and lung tissues was determined using mice ELISA kits and the manufactures’ instructions.

### 2.8. Lung tissue H&E staining

Lung tissues were fixed in 4% paraformaldehyde for at least 24h. Lung tissues fixed in paraffin were sliced into slices (5 μm), and then slices were deparaffinized in xylene and rehydrated in 100%, 95%, 85% and 75% ethanol. Haematoxylin and eosin were used to stain them. The slices were discovered using a light microscope (Leica, Germany)

### 2.9. Immunofluorescence lung tissue staining

The lung tissue slices were prepared according the description in 2.8. After blocking, the slices were stained with the primary antibody of p-PI3K, p-AKT, E-cadherin or Vimentin at 4 °C. The secondary antibody and DAPI were stained in dark. The fluorescence photographs were obtained using a fluorescence microscope (Leica, Germany).

### 2.10. Statistical analyses

Each group had ≥ 6 mice, while all tests were repeated in triplicates. The date was shown as mean ± standard error (SEM). Statistical analyses were conducted with one-way ANOVA by GraphPad Prism (ver 5.0; La Jolla, CA, USA) and *p* value <.05 was regarded as a statistical significance.

## 3. Results

### 3.1. TNBC metastasis suppression by cGAMP

**Figure 1.**
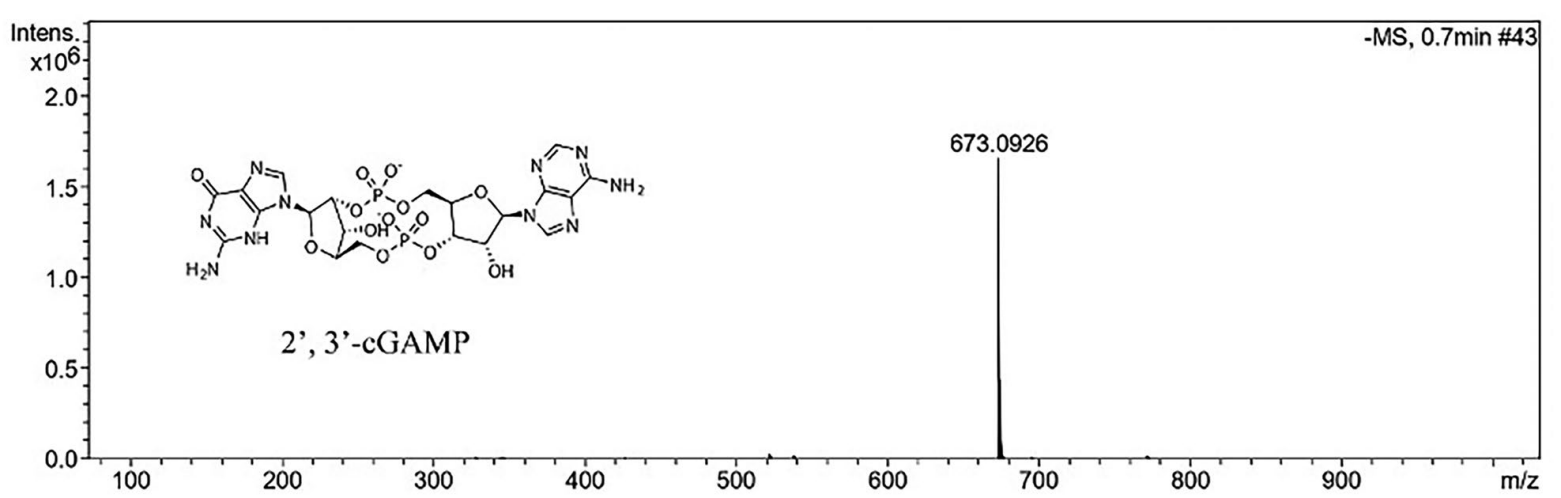
The chemical structure and mass spectrum of 2’,3’-cGAMP molecule.

The mass spectrum data regarding successful cGAMP synthesis are inferred in Figure 1. Among the mouse TNBC models, 4T1-Luc cells in BALB/c mice were widely used in the current study because 4T1 cells metastasis spontaneously to locations influencing in human BC (e.g. liver, lung and even bone) in an immunocompetent host [18,19]. *In vivo* imaging technologies were used to investigate particular biological and cellular processes in living animals using the reporter gene methodology [20]. When the firefly luciferase gene was expressed, luciferase was produced, which converted the substrate D-luciferin to non-reactive oxyluciferin, resulting in the emission of green light at 562 nm [21].

**Figure 2.**
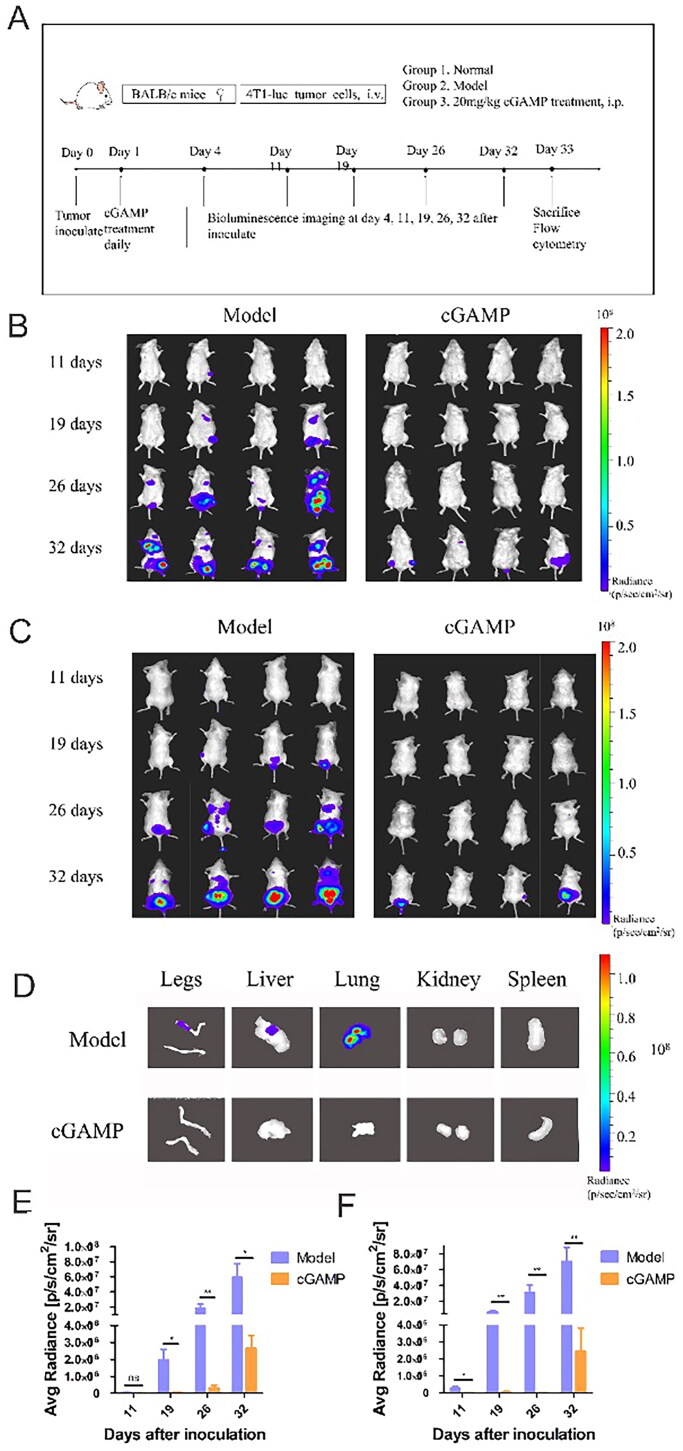
Bioluminescence imaging of tumour growth was monitored during whole experiment. (A) Schematic diagram of experiment process. Female BALB/c mice tail veins were injected intravenously with 4T1-Luc cells. During 32 days, the whole animals were imaged, and images were observed from abdomen (B) and back (C). At the end of the administration, the organs and hind limbs (D) were removed and scanned. The correlation between colour and light radiance was shown by colour bar on right side of the figure. The radiance efficiency was quantified from back (E) and abdomen (F) by Living Imaging software. Representative results were given from three experiments conducted on six mice per subgroup. Results were analysed by GraphPad Prism. Data were represented by mean ± SEM, *p < .05, **p < .01 and ***p < .001.

4T1-Luc cells in exponential phase of proliferation were collected to inoculate into female BALB/c mice by tail vein. Daily intraperitoneal administration of 20 mg/kg cGAMP was started on the two days after tumour inoculation. On the 4 days, 11 days, 19 days, 26 days and 32 days, mice were injected with luciferin, and the tumour proliferation and metastasis in mice were monitored by animal bioluminescence imaging after anaesthesia (Figure 2(A)).

Tumour cells metastasized through blood circulation and formed new tumours in thoracoabdominal region (Figure 2(B)) and back region (Figure 2(C)). The aggressiveness of tumour cells and the amount of tumour cells in the mice’s bodies grew with time. Imaging of visceral organs and hind limbs advised that the tumour metastasized to some sites without treatment, such as liver, lung and bone (Figure 2(D)). However, with administration of cGAMP every day, tumour metastasis could be inhibited. At the end of the experiment, we observed only a very small number of tumour cells, and after dissection, the tumour cells could not be trapped in the primary organ. What’s more, the region of interest (ROI) was selected around the signals to analyse photometry. Tumour-bearing mice treated with cGAMP achieved the lowest mean radiance of tumour mass at the experiment endpoint, which was verified by IVIS (Figure 2(E,F)). The findings demonstrated the efficacy of cGAMP treatment for metastatic tumour suppression, including tumour size and growth in organs. After being injected into the vein, the tumour cells were transported throughout the body, simulating tumour cell metastasis. Tumour cells may be found in several organs during blood circulation. Intraperitoneal cGAMP injection may overcome the disadvantage of intratumoural injection and be advantageous to cancer metastasis treatment.

### 3.2. Improvement of body weight and splenomegaly by cGAMP

**Figure 3.**
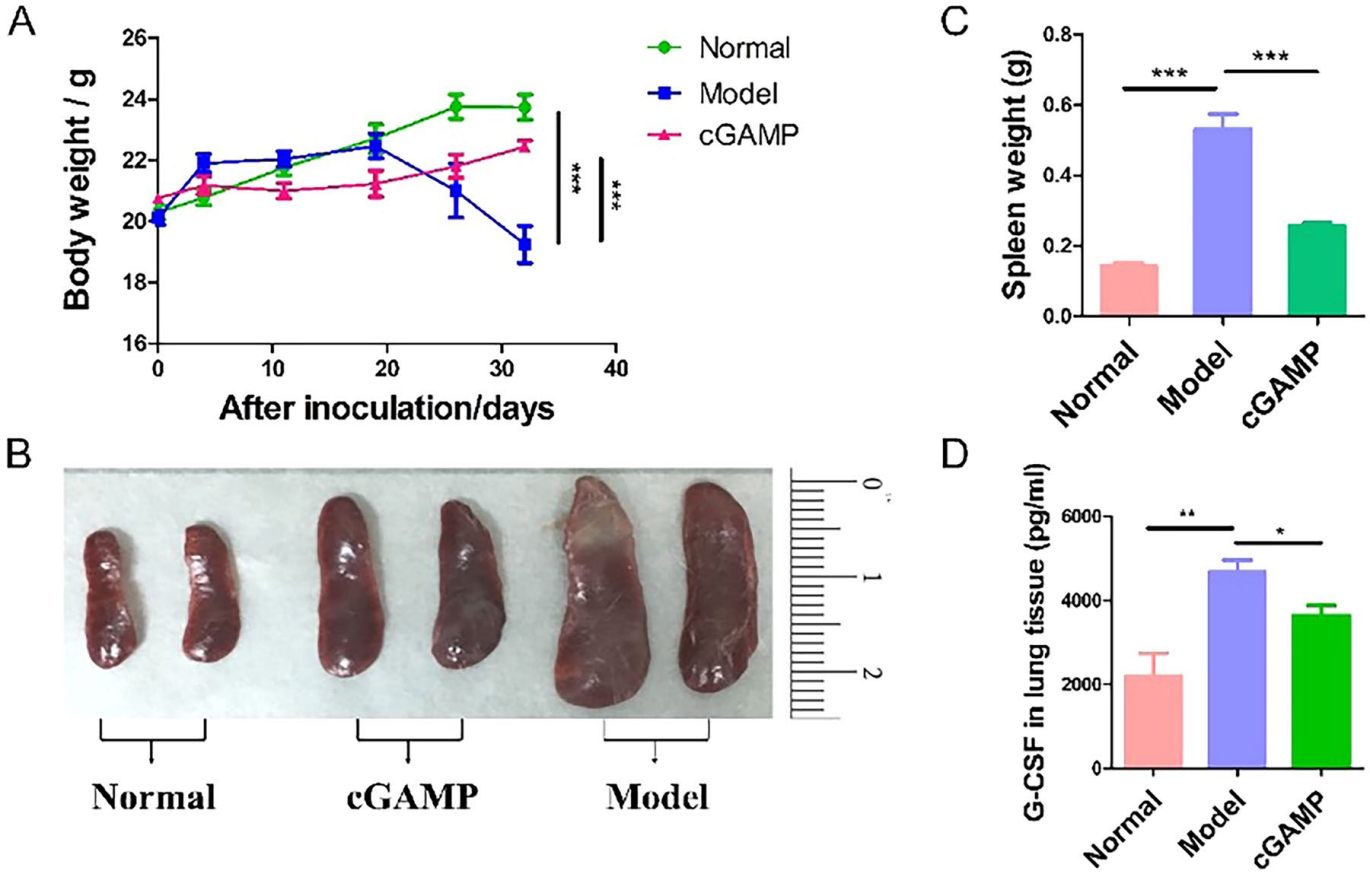
The variation of body weight and spleen. (A) Variation trends of body weight of three groups of mice during the administration. (B) Spleens isolated from sacrificed mice on 33 days after inoculation. (C) Spleen weight of mice on 33 days post-inoculation. (D) The G-CSF expression level in serum. Representative data were showcased from three experiments conducted on six mice per group. Data were analysed by GraphPad Prism. Data were represented by mean ± SEM, *p < .05, **p < .01 and ***p < .001.

**Figure 4.**
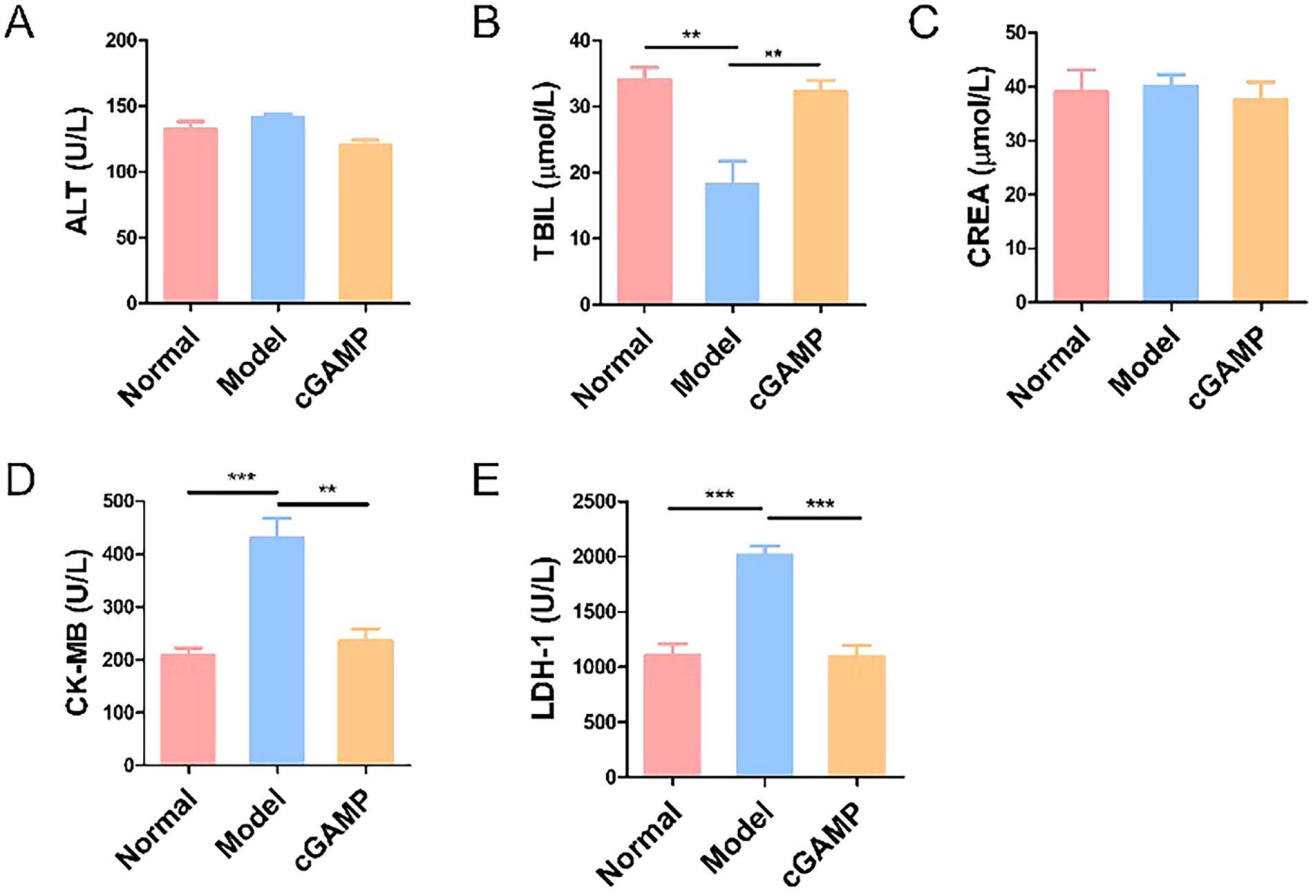
Serum biochemical test of mice. Three trials with six mice per group were demonstrated with representative data. The data were reported as mean ± SEM, *p < .05, **p < .01 and ***p < .001.

It was obviously found that the weight of mice bearing tumours decremented quickly since 20 days after inoculation, but the other groups of mice grew normally (Figure 3(A)). The weight of the animals was measured on a regular basis as an indirect indicator of toxicity. Mice treated with cGAMP didn’t exhibit body weight loss, which meant cGAMP administration with 20 mg/kg was tolerable in mice. In addition, the concentrations of ALT, TBIL, CREA, CK-MB and LDH-1 in the serum of mice were determined at the end of this study (Figure 4). The concentrations of ALT and CREA were not significantly different in the three groups, indicating that the liver and kidney functions of the mice in each group were not impaired. The TBIL concentration in the Model group mice was lower than in the other two groups, which was consistent with the Model group mice’s low RBC and haemoglobin levels. The higher concentration of CK-MB and LDH suggested that the myocardium and the cardiopulmonary function of mice in the model group were damaged, which may be due to the lung metastasis of cancer cells. Although the hearts of mice treated with cGAMP functioned normally, there was no evidence of harm from cGAMP. Meanwhile, the blood routine examination of mice treated with cGAMP were all within the normal range (Table 1). These results demonstrated that the cGAMP therapy didn’t induce adverse events. Some mice’s major organs including lung and liver were damaged by invasion and metastasis of tumour cells, which affected mice’s respiratory, metabolic and immune systems. On 32th day, the mice were sacrificed to remove spleens. Spleens of mice injected with 4T1-Luc cells weighed > 0.5 g on average (Figure 3(B,C)). By comparison, the spleens of normal and cGAMP-administration groups were less than 0.2 g and 0.3 g, respectively. Splenomegaly in tumour-bearing hosts was a marker of granulocyte and myeloid cell growth caused by cancer-related inflammation [22]. Splenomegaly in mice with 4T1-Luc tumours might be due to the high quantity of granulocyte colony stimulating factor (G-CSF) produced by 4T1 cells (Figure 3(D)). G-CSF was a type of glycoprotein that was produced by monocytes and macrophages activated by endotoxin, TNF-α and IFN-γ, which acted on differentiation, proliferation and activation of neutrophil lineage haematopoietic cells and stimulated maturation of granulocytes and mononuclear macrophages [23]. Therefore, the splenomegaly and high level of G-CSF suggested angiogenesis and tumour metastasis in model group. Our results demonstrated that cGAMP treatment could improve living state of tumour-bearing mice, reduce the secretion of G-CSF by 4T1 tumour cells and alleviate the phenomenon of splenomegaly, which may be beneficial to tumour metastasis inhibition.

**Table 1.** Blood routine examination of mice.

Group	WBC	Gran #	RBC	HCT	PLT	HGB
Model	10.2 ± 1.3	8.7 ± 0.5	4.6 ± 1.3	27.5 ± 3.0	556.0 ± 109.4	83.1 ± 4.5
cGAMP	5.8 ± 0.4	1.5 ± 0.2	9.2 ± 1.8	40.2 ± 4.1	1254.0 ± 88.7	135.0 ± 5.2
Reference ranges	0.8–6.8 (10^9/L)	0.1–1.8 (10^9/L)	6.3–9.4 (10^12/L)	34.6–44.6%	450.0–1590.0 (10^9/L)	110.0–143.0 g/L

*Note:* WBC: white blood cells; Gran #: neutrophil count; RBC: red blood cells; HCT: haematocrit; PLT: platelet; HGB: haemoglobin.

### 3.3. Reduction of pulmonary metastasis by cGAMP

**Figure 5.**
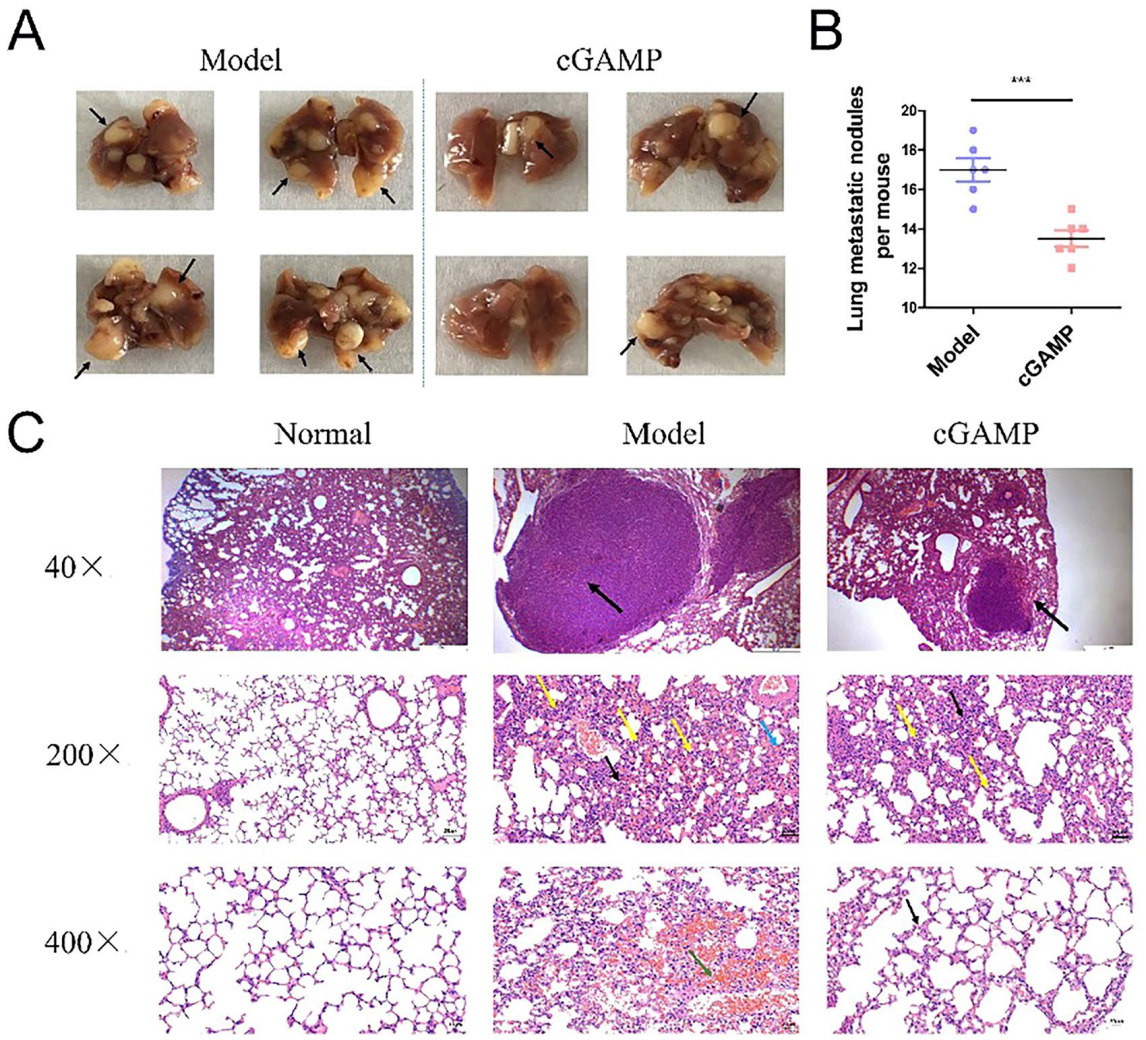
Pulmonary metastasis of 4T1-Luc BC cells. (A) Tumours in lung tissues were extracted from the bodies of deceased mice, which were preserved in 4% paraformaldehyde for at least one day. (B) Pulmonary metastatic nodules per mouse, each dot represents one mouse. (C) Assay for haematoxylin and eosin staining of paraffin-embedded lung tissues. Illustrative results were provided from three experiments performed on six mice per subgroup. GraphPad Prism 5.0 was used to analyse the data. The data were reported as mean ± SEM., *p < .05, **p < .01 and ***p < .001.

TNBC has a high rate of metastasis and tends to metastasize to lung tissue with some capillaries. The lung tissues of sacrificed mice were obtained and fixed in 4% paraformaldehyde for at least one day. Pulmonary metastasis nodules were visualized after morphologic fixation, which were counted manually (Figure 5(A)). The average number of metastatic tumour nodules in lung tissues was 17 in the model group, but 13 in the cGAMP-administration group (Figure 5(B)). In the H&E staining results, the nuclei and fragments of tumour cells in the tumour tissue were stained blue-purple, but the cytoplasm was pink (Figure 5(C)). Results showed that the lung tissues of normal mice were intact structurally. The alveolar cavities were clear and the alveolar septum had no distinct manifestation such like oedema or inflammation. On the contrary, several large tumour nodules, extensive alveolar wall thickening (200×, black arrows), narrow alveolar cavities, multiple alveolar haemorrhages (green arrow), more capillaries and vascular congestion (blue arrows) and infiltration of monocytes and neutrophils (yellow arrows) were present in lung tissue of the mice model. Occupation and deformation of blood vessels and alveolar cavities could cause dyspnoea, which led to death in mice. The frequency and size of metastatic nodules in the lungs of cGAMP-treated mice were dramatically decreased, and only tiny tumour nodules were observed. In mice lung tissues, the lung parenchyma was a large number of alveoli at various branches of bronchial tubes and their terminals. The epithelial cells of bronchial tubes were closely arranged without obvious degenerations and necrosis or exfoliation that observed. A few alveolar walls were slightly thickened (400×, black arrows) with few infiltrations of mononuclear cells and neutrophils (yellow arrows). The results informed that cGAMP treatment could suppress metastasis and growth of BC cells in lung tissues, which maintained normal mice lung function and improved the survival rate of mice.

### 3.4. Immune cells activation in spleen by cGAMP

**Figure 6.**
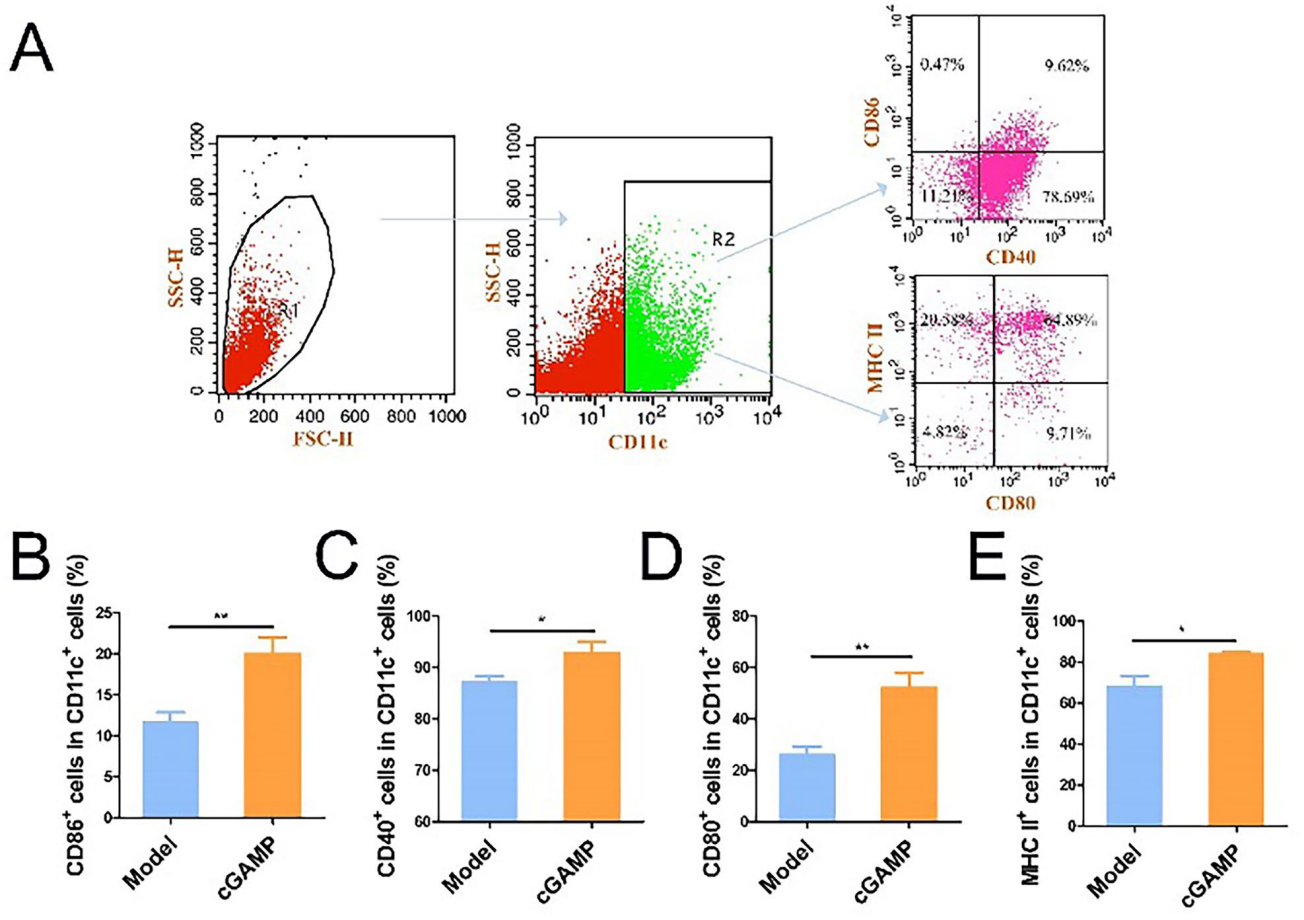
Flow cytometry analysis regarding spleen immune cells. (A) Illustration of a gating technique for detecting CD80, CD40, MHCII and CD86 in spleens. (B–E) The percentages of viable cells for CD80, CD40, MHC II and CD86 of DCs in spleens. Three studies were presented with representative results from six mice per group. GraphPad Prism was used to analyse the data. The mean ± SEM was used to depict the data, *p < .05, **p < .01 and ***p < .001.

**Figure 7.**
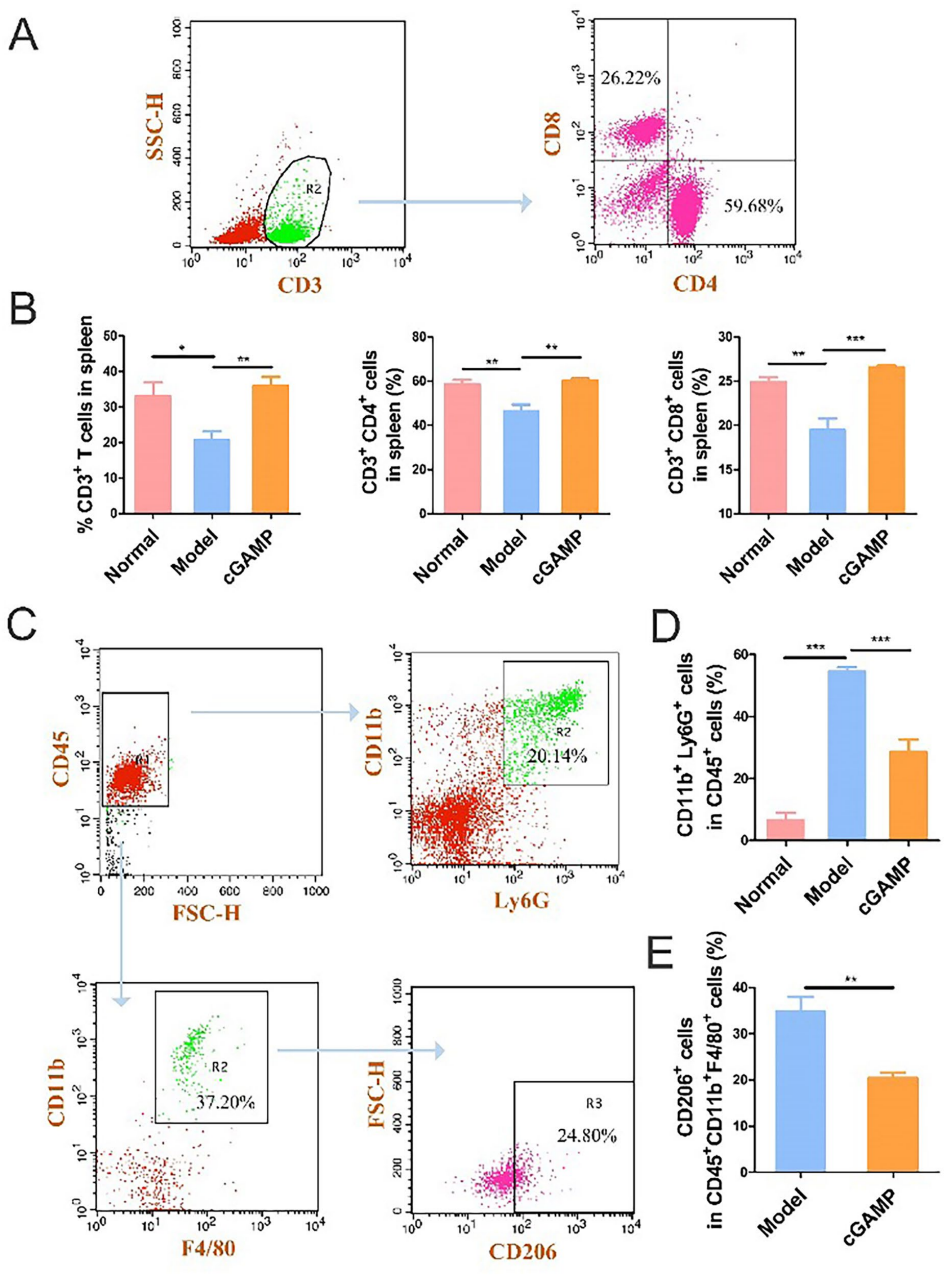
Flow Immune cells in the spleen were analysed using cytometry. (A) Illustration of a gating method for detecting CD3, CD4 and CD8 in spleens. (B) Percentage of CD3, CD4 and CD8 in spleens. (C) A typical gating technique for detecting M2-TAM and Granulocytic MDSC in spleens. (D) Neutrophil count in spleens expressed as a proportion of viable cells. (E) M2-TAM in spleens was estimated as a proportion of viable cells. Three trials with six mice per group were demonstrated with representative data. GraphPad Prism was used to analyse the data. Data were represented by mean ± SEM, *p < .05, **p < .01 and ***p < .001.

Dendritic cells (DCs) were antigen-presenting cells (APCs) *in vivo*, playing critical roles in the regulation of innate and adaptive immunity [24]. Surface co-stimulatory markers were found at modest amounts in immature DCs. Previous study has shown that cGAMP may directly activate DCs *in vitro* [25]. In the detection experiment of immune cells, CD11c^+^ cells were gated to examine the expressions of CD40, CD86, CD80 and MHC II. cGAMP treatment dramatically increased the expression of these four DC markers (Figure 6), indicating that DCs developed in response to the second messenger cGAMP activation. cGAMP stimulated DC to create IFN-β *via* the cGAS-STING-IRF3 pathway and IFN-β then cross-primed tumour-specific CD8^+^ T cells [26–28]. Because CD8^+^ T lymphocytes could efficiently destroy tumour cells and CD4^+^ lymphocytes aided the immune system in its fight against microorganisms, the amount of CD8^+^ and CD4^+^ T cells was greater in the cGAMP-treatment group (Figure 7(A,B)).

Tumour associated macrophage was expressed highly amongst most cancer patients, who suffered from promoted tumour proliferation and metastasis [29]. In the early cancer stage, M1-type TAMs had a killing effect on tumour cells. With the cancer development, TAM was polarized to M2-type TAMs, which was conducive to tumour proliferation. By labelling specifically M2-type TAMs , the CD206 expression level incremented in model group and decreased in spleens of mice in the cGAMP administration group (Figure 7(C,E)). cGAMP therapy could reshape the tumour microenvironment to play an anti-tumour effect.

Granulocytic MDSCs, defined as CD45^+^CD11b^+^Ly6G^+^ cells, were the major components of non-specific immunity. They could not only resist microorganisms and pathogens, but also function importantly in tissue repair [30]. However, some investigations illustrated that neutrophils could affect proliferation, migration and metastasis of malignant tumours in multiple ways [31]. What’s more, neutrophils suppressed CD8^+^ T cell activation to facilitate metastasis of many cancers, especially BC [32]. In BC patients, increased neutrophils predicted a worsening of metastasis-specific survival [33,34]. The presence of tumours significantly increased the expression level of neutrophils in model group mice and induced the metastasis of cancer cells to the whole body of mice. In contrast, neutrophils decreased in the cGAMP-treated mice, resulting in a significant reduction in lung and lymph node metastasis (Figure 7(C,D)).

### 3.5. cGAMP induced cytokines to inhibit tumour metastasis

**Figure 8.**
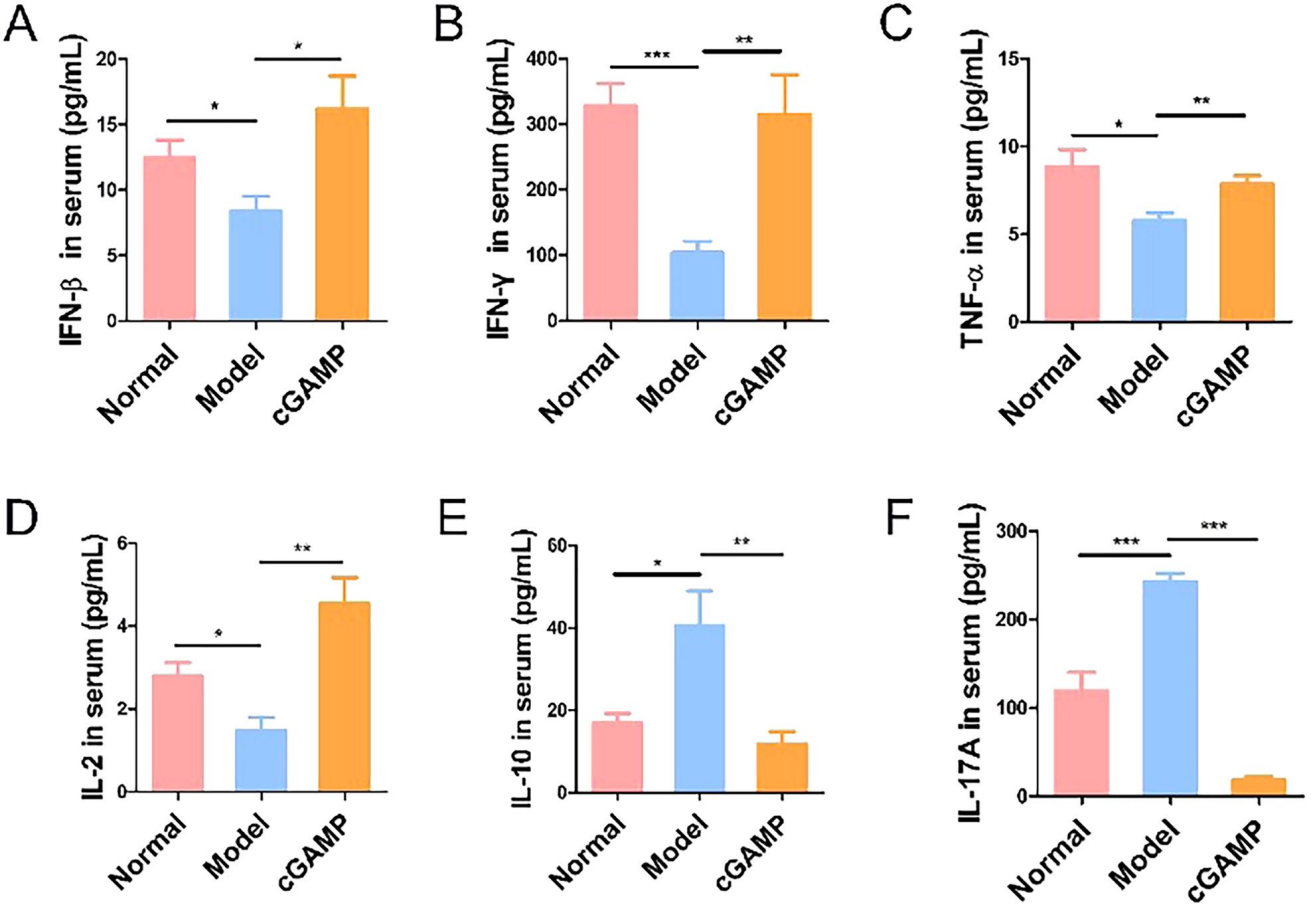
The expression of IFN-β, IFN-γ, TNF-α, IL-2, IL-10 and IL-17A cytokines in serum were measured by ELISA. Three trials with six mice per group were demonstrated with representative data. Data were analysed through GraphPad Prism. Data were represented by mean ± SEM, *p < .05, **p < .01 and ***p < .001.

ELISA was used to detect antitumour cytokine expression in serum. In comparison to tumour-bearing animals, cGAMP treatment stimulated the production of immune cytokines with anticancer potential, such as IFN-β and IFN-γ (Figure 8(A,B)). Endogenous type I IFN served as innate cells’ initial line of defence, increasing adaptive immune response against viruses and cancer cells [35,36]. STING is widely expressed in cells and can be activated by pathogens or damaged cell’s cellular nucleotides to initiate signalling pathway. Intratumoural injection of synthetic cyclic dinucleotides (CDNs) in mice has been demonstrated to rapidly induce IFN-β production in intra-tumour immune cells and create anti-tumour-specific CD3^+^CD8^+^ T cells that limit tumour development [37,38]. Increased IFN-β levels imply that cGAMP binds to STING as a secondary message, activating the cGAS-STING-IRF3 pathway and stimulating the innate immune response. Meanwhile, IFN-β may activate tumour cells and cause tumour cell death *via* the tumour necrosis factor-related apoptosis-inducing ligand (TRAIL) pathway [39].

Tumour necrosis factor-α (TNF-α), mainly generated by activated mononuclear macrophages and other cells, was a cytokine capable of causing haemorrhagic necrosis of tumour tissue cells [35]. The up-regulation of TNF-α may prime cancer cells towards apoptosis (Figure 8(C)). Mechanistically, TNF-α induced autophagy and prevented energy transfer from the tumour microenvironment [40]. IL-2 was a powerful immune growth factor and played an indispensable role in sustaining T cell response [41], which also increased under drug delivery (Figure 8(D)). It was mainly secreted by CD4^+^ T cells being known as antigen stimulation response [42], which was consistent with elevated CD4^+^ T cells expression by flow cytometry test. Purified IL-2 infusion significantly increased lymphocyte persistence and anticancer impact in mice [43,44]. Rosenberg and colleagues demonstrated that giving mice recombinant IL-2 resulted in substantial anticancer action, including the regression of existing lung metastases and subcutaneous tumours [45].

On the contrary, the current study revealed that elevated serum levels of IL-10 and IL-17A in tumour-bearing mice were strongly associated with BC metastasis, and cGAMP treatment could counteract the effect (Figure 8(E,F)). Interleukin-10 (IL-10) was initially heralded as a major immune suppressive factor through the suppression of TH1 immune response and T cell cytotoxic activity [46,47]. IL-10 inhibited effector T cell proliferation, cytokine generation and migration, allowing tumour cells to avoid immune detection [46]. G-CSF and IL-17A serum levels were greater in tumour-bearing mice than in normal mice. IL-17 produced by gamma delta (γδ) T cells resulted in systemic G-CSF-dependent neutrophil proliferation and polarization in mice with mammary tumours, correlating with earlier experimental findings. Tumour-induced neutrophils were found to be capable of inhibiting CD8^+^ T cells, hence promoting tumour development and metastasis [32]. The findings reveal that breast cancers cause γδ T cells to release IL-17A, which causes systemic proliferation and polarization of neutrophils to the CD8^+^ T cell suppressor type, followed by the establishment of breast tumour metastasis in distant organs. Certain cytokines can be modulated by cGAMP treatment to exert anti-tumour action and prevent tumour spread.

### 3.6. Mechanisms for tumour metastasis suppression

Certain chemokine receptors on tumour cell surfaces were involved in multiple steps of tumour genesis and metastasis [48]. CXCR4 was found on the surface of murine breast tumour 4T1 BC cells, and it was found to increase breast tumour development and metastasis when combined with the chemokine CXCL12 (SDF-1) [49–51].

**Figure 9.**
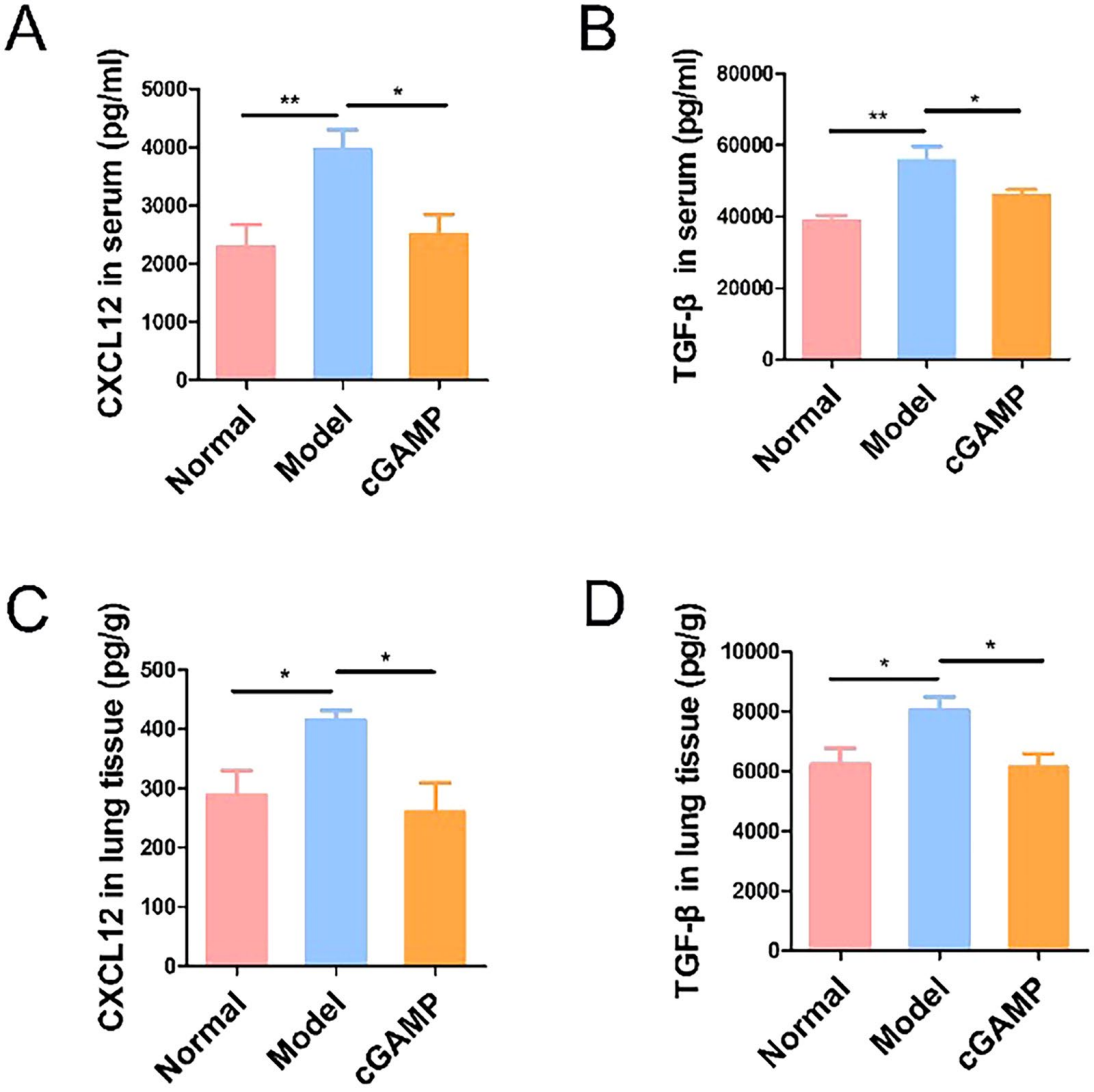
The production of TGF-β and CXCL12 were measured by ELISA in serum and lung tissues. Representative data were shown from three experiments conducted on six mice per group. Data were analysed by GraphPad Prism 5.0. Data were represented as mean ± SEM, *p < .05, **p < .01 and ***p < .001.

In our experiments, the cytokines including TGF-β and CXCL12 in serum and lung tissues were measured by ELISA to analyse the impact on BC metastasis. TGF-β, highly generated by tumour cells, and some suppressive immune cells, could induce tumour cells metastasis by the EMT (epithelial-mesenchymal transition) process [52]. As a result, TGF-β expression was greater in animals containing breast tumour cells than in normal mice, and cGAMP therapy may diminish it to limit breast tumour cell migration (Figure 9(B)). TGF-β was frequently accumulated in tumours where it could influence the activation and differentiation of DCs [53]. TGF-β in tumour microenvironment may limit the synthesis of pro-inflammatory immune factors such as TNF-α, IFN-γ and IL-12, while promoting the release of anti-inflammatory factors such as IL-10 [54,55]. As a result, TGF-β expression in lung tissue was increased in tumour-bearing mice (Figure 9(D)).

MSCs (mesenchymal stem cells) were pluripotent stem cells that enable to produce several chemokines, such as CCL5 and CXCL12 [56]. CXCL12 has been broadly researched in BC metastasis [57]. We also confirmed that more CXCL12 was expressed in serum of breast tumour-bearing mice (Figure 9(A)). Many organs with high CXCL12 expression levels were related with common locations of BC cell metastasis [14], which was compatible with experiment results. The CXCL12 expression level was higher in neoplastic lung tissue, which was occupied with breast tumours (Figure 9(C)).

**Figure 10.**
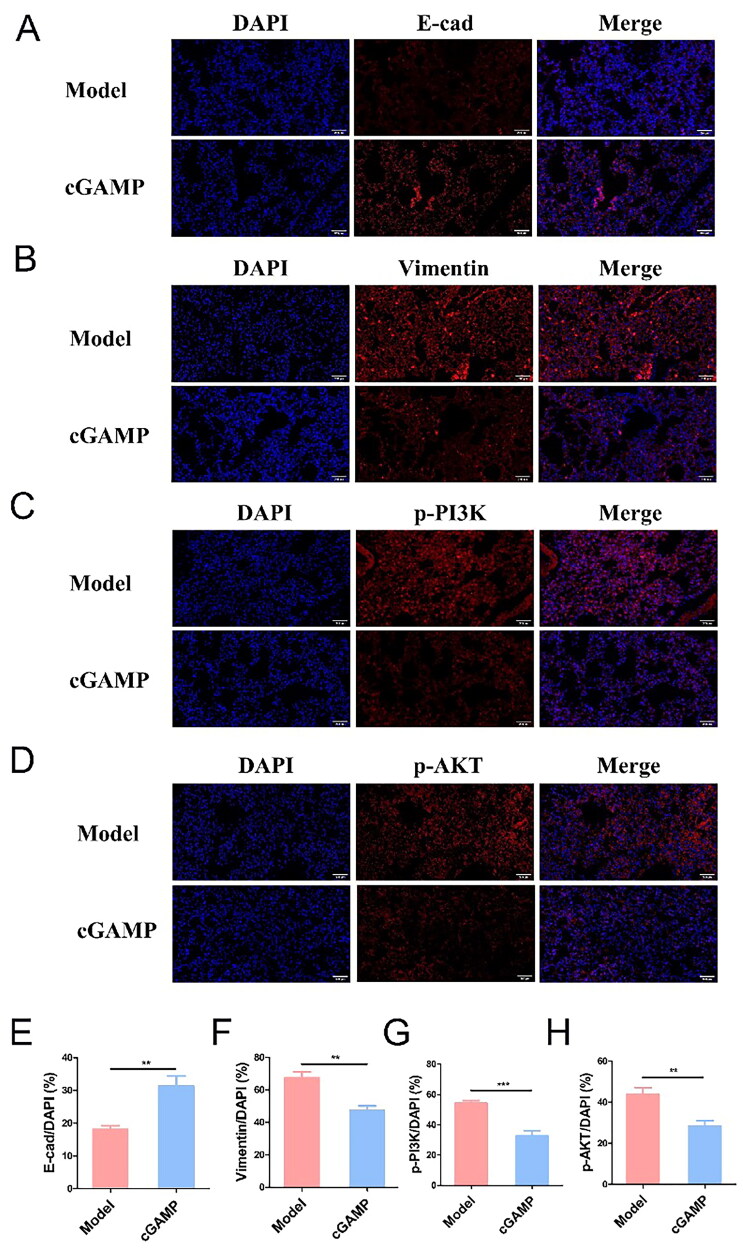
The expressions of E-cad (A), Vimentin (B), p-PI3K (C) and p-AKT (D) in mouse lung tissues were detected by immunofluorescence (magnification = 400×). The semi-quantitative calculation of fluorescence images (E–H). Three trials with six mice per group were demonstrated with representative data. Data were analysed by GraphPad Prism 5.0. Data were represented as mean ± SEM, *p < .05, **p < .01 and ***p < .001.

Furthermore, the EMT process has been extensively researched as one of the causes of tumour spread. The EMT process, which is driven by essential transcription factors, allows epithelioid tumour cells to adopt a mesenchymal character, increasing their propensity to spread. E-cadherin (E-cad) was found in high concentrations on epithelioid cells, whereas Snail, Twist and Vimentin were found in high concentrations on stromal cells [58,59]. EMT process could be regulated by TGF-β, and other experiments have verified that cGAMP regulated EMT process through Wnt/β-catenin by itself [60]. The immunofluorescence analysis in the lung tissues clearly showed that the cGAMP treatment yielded a high E-cad level and low Vimentin level (Figure 10(A,B)), which consequently demonstrated that cGAMP was beneficial to the transformation of metastatic mesenchymal to epithelioid cells due to restricted expression of TGF-β in tumour microenvironment, thereby inhibiting the EMT process. In addition, it could be observed that phosphorylated proteins p-PI3K and p-Akt were highly expressed in lung tissues of mice in model group (Figure 10(C,D)), indicating that this pathway was activated in tumour cells to promote tumour metastasis, which was further illustrated by semi-quantitative statistics of fluorescence intensity (Figure 10(E–H)).

CXCR4 binding to the ligand SDF-1 activated the phosphoinositide 3-kinase/protein kinase B (PI3k/Akt) signalling pathway [14], which had a role in the initiation and development of BCs and regulated a range of cellular processes such as survival, proliferation and metabolism [61,62]. TGF-β, secreted by M2-TAM in the tumour microenvironment, was recognized as a potent immunosuppressor, which accumulated in the tumour microenvironment, specifically interfered with the phosphorylation of IRF3, thus inhibited type I IFN response, suppressed the function of immune effectors and promoted tumour growth and metastasis [63]. The TGF-β and CXCL12 expression levels in tumour-bearing mice were significantly higher than those in normal mice, explaining metastasis regarding tumour cells to multiple organs. TGF-β not only induced tumour cell migration through the EMT process, but also increased the CXCR4 expressions in 4T1 tumour cells, which combined with CXCL12 to activate intracellular PI3K/Akt pathway, promoting the occurrence and metastasis of breast tumours [64].

**Figure 11.**
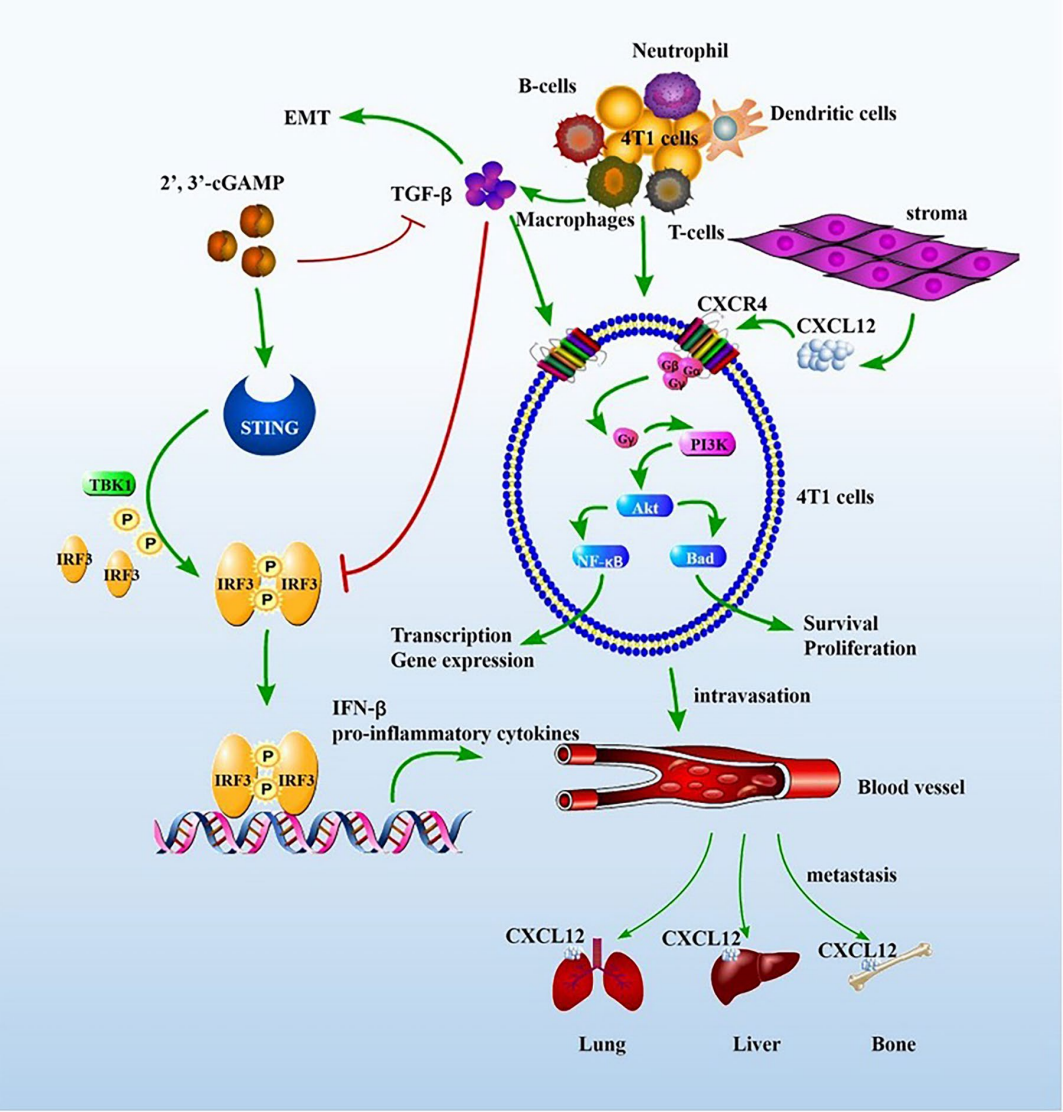
The proposed mechanisms for tumour metastasis suppression. The schematic pathway was drawn by Pathway Builder Tool 2.0.

Based upon immune cytokine expressions in mice serum and lung tissues, the expression of immune cells in spleens and the regulation of related proteins in PI3K/AKT signalling pathway and EMT signalling pathways, the inhibition mechanism of BC pulmonary metastasis was summarized as follows: cGAMP administration could activate STING immune pathway, restore IRF3 phosphorylation and induce IFN-β production, which activated innate immunity and adaptive immunity response, promoted CD8^+^ T cell proliferation and produced IFN-γ. cGAMP could reshape tumour microenvironment, reverse the immune-suppressive microenvironment caused by M2-TAM and reduce the polarization of M2-TAM, thus decreased the expression of TGF-β secreted by M2-TAM, inhibiting the EMT process and tumour metastasis. The reduction of TGF-β, which reduced CXCR4 receptor expressions on the tumour cell surfaces and weakened the intracellular PI3K/AKT pathway activation, further impeded tumour development and metastasis (Figure 11).

## 4. Discussion

Breast cancer is a super common cancer type affecting female in the globe, whose morbidity and mortality are increasing. The Breast cancer development and occurrence are relevant to multiple signalling pathways, such like the PI3K/AKT/mTOR pathway, Ras/Raf/MEK pathway, JAK/STAT pathway and cell cycle checkpoint, and it is of importance to study synergy effects of multiple pathways. STING agonists are widely applied for tumour immunotherapy. CDNs, as a kind of STING agonist, can activate the STING signalling pathway directly. It was found that CDNs had potential anticancer activity to inhibit the proliferation of human cancer cells [65]. Most clinical trials of STING agonists were administered by direct intratumoural injection. Intratumoural injection of 2′,3′-cGAMP led to the increase of TAMs in tumour tissues and polarized M2-TAM to M1-TAM, re-modeling the tumour microenvironment in many tumour types, including 4T1 mouse breast cancer, CT26 mouse colon cancer and B16F10 mouse melanoma. In the B16F10 mouse model, intratumoural injection of 2′,3′-cGAMP might significantly delay tumour growth and reduce lung metastases [37]. Systemic administration of 2′,3′-cGAMP could also inhibit the tumour growth of colorectal cancer in mice [66]. STING agonists are also used in cancer immunotherapy in conjunction with immune checkpoint inhibitors. The combination of the STING agonist ADU-S100, the PD-L1 inhibitor, and the OX40 agonist not only activated the immune system to increase T cell activation, but it also overcame immune tolerance [67]. Some anticancer cGAS-STING agonists, such as ADU-S100 (MIW815), MK-1454 and E7766, have been cleared for clinical studies in humans to assess their capacity to mediate cancer progression [68]. However, Corrale et al. highlighted that optimal drug levels can be achieved by direct intratumoural administration, but this limited their applications in metastatic and systemic tumours [38]. Systemic distribution of the drug may be beneficial to prevent cancer cell metastasis in the whole body. Other non-nucleotide-based STING ligands were also synthesized, which are not limited to intratumoural administration. For example, MSA-2, which was subcutaneously and orally available in mice, was well tolerated and induced tumour regression with durable antitumour immunity [69].

In our current investigation, we tested the antitumour effect and toxicity of cGAMP by systematic administration in TNBC. The findings revealed that cGAMP activated innate and cross-primed adaptive immunity, increased and enhanced antigen-presenting activity of DCs, and activated CD8^+^T cells to effectively destroy tumours and reversed inhibitory immune microenvironment. It was worth noting that neutrophil and TAM, associated with metastasis, decreased in the treatment group. The bone marrow monocytes circulating in the blood differentiate into macrophages and become an important part of innate immunity. Monocytes enter tumour tissues and differentiate into tumour-associated macrophages (TAMs) under tumour microenvironment influence. The number of neutrophils and TAMs in patients is connected to tumour progression and patient survival, and cancer cells can hijack the host’s protective immune system, promoting further growth and metastasis [31]. Upon activation, neutrophils release a fibrous network-like structure named neutrophil extracellular traps (NETs), which are abundant in a variety of malignant tumours [70–72]. Currently, more pieces of evidence demonstrate that NETs can promote tumour progression and metastasis by destroying vascular integrity [73,74]. cGAMP-administration reduced the expression of neutrophils and TAM to protect the immune system, resulting in a significant reduction during lymph node and pulmonary metastasis.

We also explored the mechanisms for tumour metastasis suppression of cGAMP in detail. Increasing evidence suggested that many tumour progression steps were influenced by the tumour microenvironment. Cancer cells were impacted not only by each other, but also by extracellular matrix (ECM) and other microenvironmental cells such as endothelial cells, fibroblasts and inflammatory cells [75].

CXCL12/CXCR4 signal transduction not only promotes dysplasia and stress of tumour tissues, inducing physical barrier for therapeutic agents and T cell penetration, but also functions importantly to promote tumour cell migration, proliferation and invasions [76,77]. CXCR4 is highly expressed in tumour cells and tumour-associated fibroblasts (TAF) of various cancers [78,79]. The up-regulation of CXCR4 has been linked to tumour development, angiogenesis, invasion and migration [80–82]. Stimulation of CXCR4 in the cytoplasm of BC cells induces nitrous oxide and is associated with lymphoid node shift [83]. More importantly, the expression of CXCR4 is a prognostic marker for BC [84]. BC has distinct metastatic patterns that are unrelated to blood flow patterns, showing that cancer cells preferentially homing, adhesion, survival and/or proliferation in certain organs and tissues. CXCL12 functions critically as chemoattractant in cancer progression and invasion of malignant tumours [85]. CXCL12 is constitutively released in various organs, including the lung, brain, liver, skeletal muscle, heart, skin, kidney and bone marrow, and it promotes ischaemic tissue revascularization and tumour development [86,87]. Therefore, CXCR4^+^ tumour cells tend to metastasize to organs that express CXCL12 *via* blood vessels [88]. Pharmacological blocking of CXCR4 or CXCL12 has given encouraging anticancer effects [89–91]. Some small molecule inhibitors and monoclonal antibodies are being developed to inhibit the interaction between CXCR4 and CXCL12. However, therapeutic effectiveness of the therapies is limited by speedy clearance *in vivo*, poor tissue permeability and systemic toxicity [92].

STING is expressed ubiquitously in cells, and cytosolic nucleotides from pathogens or damaged cells could activate STING. Particularly, it is showcased that intratumoural injection of synthetic cyclic dinucleotides (CDN) could elicit rapidly the IFN-β expression by immune cells in tumours and generate anti-tumour specific CD8+ T cells to inhibit tumours development [93]. TGF-β is secreted by immune cell lineages such as B cells, T cells, dendritic cells and macrophages, which adversely controls their proliferation, differentiation and activation in the advanced tumours [37]. The TGF-β accumulation in tumour microenvironment prevents production of IFNα/β after STING activation, inhibits the function of immune effectors and accelerates metastasis and diffusion [94]. According to Ao et al. increased stromal TGF-β may induce the expression of epithelial CXCR4, which allowed stromal SDF-1 to activate the PI3K/AKT pathway in epithelial cells, leading to tumour development and predisposing cells to further malignant progression [64]. cGAMP could play a synergistic effect to inhibit tumour growth and metastasis by targeting simultaneously multiple immune pathways.

While immunotherapy is extremely promising, one major difficulty is that many malignancies continue to be resistant to existing cancer treatment. This study demonstrated the exceptional efficacy of cGAMP alone on TNBC treatments, despite the fact that most other medications showed no response. T cell response and antitumour memory were evoked by cGAMP, which inhibited the growth of metastatic cancers in organs. The findings revealed that cGAMP might achieve an effective antitumour immune response in cancers that had limited or no response to immune checkpoint targeted therapies. As a result, cGAMP might represent a unique potential TNBC immunotherapy.

## 5. Conclusions

This study found that cGAMP treatment can decrease breast tumour metastasis and, in particular, diminish pulmonary metastasis, increasing mice’s living conditions and survival rate. As the second message, cGAMP, might activate innate and adaptive immunity in mice *via* the cGAS-STING-IRF3 pathway, thereby triggering the generation of cytokines and immune cells to destroy tumour cells. The following mechanism was: cGAMP stimulated innate and adaptive immunity, changed the tumour immune milieu, down-regulated the expression of M2-TAM and TGF-β and impaired the combining capacity of CXCR4 and CXCL12. The tumour metastasis-facilitating pathways, including the EMT process and the PI3K/AKT pathway, were further suppressed, reducing tumour metastasis in particular organs. Based on these advantages and our findings, cGAMP might be developed as a potential immune inhibitor for the treatment of metastatic breast cancer. The innate immune and adaptive immune have complicated synergistic effects on regulating tumour metastasis. However, the mechanism of STING agonist administration that drives antitumour immune responses to suppress breast tumour metastasis remains unknown. In this study, we evaluated the antitumour efficacy of cGAMP in metastatic BC. More importantly, based upon the immune cytokine expressions in mice serum and lung tissues, the immune cells expression in spleens and the related protein regulations in PI3K/AKT signalling pathway and EMT signalling pathways, the pharmacological mechanism of cGAMP to suppress BC pulmonary metastasis was further studied.

## Data Availability

The datasets used and analysed during the current study are available from the corresponding author on reasonable request.
